# COVID-19: fighting the foe with Virchow

**DOI:** 10.1007/s15010-021-01628-3

**Published:** 2021-05-19

**Authors:** Cihan Papan, Katharina Last, Sascha Meyer

**Affiliations:** 1 Centre for Infectious Diseases, Institute of Medical Microbiology and Hygiene, Saarland University, Kirrberger Strasse, Building 43, 66421 Homburg, Germany; 2 Department of Paediatrics and Neonatology, Saarland University Medical Center, Homburg, Germany

To the editor,

We are in a fever, lying in our own emergency room with what has turned into a lingering disease.

It is grievous to concede that we are witnessing the greatest global tragedy of our generation [1]. Alas, the worst may be yet to come, with an unpredictable pandemic aftermath [2], and a plethora of zoonotic viruses harbouring the potential to cross species borders and wreak even greater havoc than COVID-19 [3]. Yet for others, these superlatives may of course seem cynical, having endured greater struggles, repeatedly—natural disasters, famines, persecution, infections or non-communicable diseases [4, 5].

The twist in this “disaster in slow-motion” [6] is that albeit predictable, COVID-19 holds us still as hostage due to our interdependence. Healthcare workers and scientists around the globe have answered with vigour and admirable resilience. Political reactions, however, have been less uplifting. Instead of concerted actions on a global scale, most countries first turned to isolationism. A more illustrative example was the provision of COVID-19 vaccines by the European Commission for its member states, which anyhow has been harshly criticized in hindsight for being irresolute, hesitant, and parsimonious [7].

On a national level, Germany may serve as a case in point. Although scientists were involved from the beginning in informing politicians, the time-to-action needed at critical inflection points—lockdown measures, vaccination roll-out, implementation of rapid testing, reaction to variants of concerns—was and still is painfully slow, owing to political processes in general, but partly also to the federal mode of governance in Germany. In addition, upcoming major elections may inadvertently bias some politicians’ behaviour, shifting away from putatively unpopular proactive measures to a politics of procrastination. As a net effect, political decision-making is delayed for several weeks, which inevitably redounds to the virus’s advantage. While the majority of the population is receptive for scientifically based and consistent approaches, the repeated and expeditiously convened meetings of the Chancellor with leaders of the federal states continue to seem like a “peri-parliamentary ping-pong” with little tangible results, causing irritation and disappointment of the public. The failure to take appropriate action creates a void that is subsequently filled, within a fortnight or two, by a growing number of hospitalized, harmed, and dead.

The liberty of each federal state in Germany to execute the infection protection act at their own responsibility, with even diverging approaches on district levels, has resembled a nationwide multi-arm, multi-stage trial on the best public health practice in tackling COVID-19. Unfortunately, translating this real-life experiment into applicable knowledge, i.e. distilling the practice from a district that has fared well and applying it elsewhere, has remained arduous at best.

Rudolf Virchow, the founding father of pathology, had put it aphoristically that "[…] politics is nothing else but medicine on a large scale” [8]. If we are the patient in our own ER, then wouldn’t it be more appropriate to take rapid, tangible, pragmatic action, without sacrificing democratic grounds in favour of a scientocracy? Wouldn’t this entail avoiding long-term complications and recurrence as well, e.g. by aiming for global vaccine equity [9]? To tackle social and economic inequities which have become painfully amplified through the pandemic? To cure not only the most obvious, but also the chronic, neglected, distant lesions, the itch between our toes, that can turn into a gangrene in a heartbeat? Wouldn’t this mean to refrain from penetrating into wildlife? To bring the climate emergency back high on the agenda? Both, COVID-19 and the climate emergency, are similarly rooted in the exploitation of the planet, and both require global, cooperative efforts to achieve sustainable goals [10].

In analogy to Virchow’s concept of pathology, the future of democracies will depend in no small part on our willingness and ability to effectively encounter and tackle problems on local (“cellular”), national (“organ”) and global (“organism”) levels, while maintaining the best balance between upholding individual rights and defending public welfare. The COVID-19 pandemic may serve as a wake-up call in doing so, since different approaches to solve this global disaster will eventually be measured not only by their means, but by their success. The many lessons that are still to be learned for a potentially much larger and more lethal, future pandemic do pertain to politicians and scientists alike. It will be paramount to delineate specific measures with regard to their effectiveness (i.e. reducing incidence, intensive care admissions, deaths), as well as to weigh them carefully against potential inherent detriment, e.g., economical or psychological (including consequences of school closures on children and adolescents). In parallel, it will be essential to develop strategies to protect from deleterious effects on human health inflicted by these very measures, e.g., impaired health care access for patients with chronic diseases, first diagnosis of cancer, and preventive medical check-ups.

Moreover, it is excruciating to realize the disproportionately higher COVID-19-related morbidity and mortality in the economically disadvantaged. The ability to practice physical distancing and to work in home-office turned out to be a privilege of the wealthy ones; those who have to make a living non-distantly will continue to put their lives at risk every day. The mitigation of this dilemma certainly needs both, political action and dedicated (public) health care as demonstrated by ad-hoc vaccine campaigns in hotspot urban neighbourhoods in the cities of Cologne and Münster.

Another aspect worth contemplating is the role of science in the public discourse. While the merits of science communication have proven invaluable in this pandemic, scientists learned steadily to acknowledge that their scientific approach is always to be integrated into a larger societal perspective. Ultimately, politicians are accountable for any measure taken or not, while the role of the scientific community is in informing both, the public and politicians.

Undoubtedly, this pandemic will ingrain in our collective memory, which may guide democratic civil societies in the future to counterbalance erratic governance, experienced during this pandemic in some countries.

In the 200^th^ year of his anniversary, it appears more than timely to commemorate Virchow, to remain politically actionable and overcome our “inearthia”, to heal us, the patient and the planet in a fever.
